# Supplementary material for: Becoming metrics literate: An analysis of brief videos that teach about the h-index
Source: PLoS One. 2022 May 6;17(5):e0268110. doi: 10.1371/journal.pone.0268110 (PMC9075661; doi:10.1371/journal.pone.0268110)
Supplement: Attachment — Submitted filename: Response to Reviewers.docx [file pone.0268110.s001.docx]

Response to Reviewers

| Comment | Response | Revision Location |
| --- | --- | --- |
| Academic Editor: |  |  |
| Please ensure that your manuscript meets PLOS ONE's style requirements, including those for file naming. | We have ensured that our manuscript meets PLOS One’s style requirements. | Throughout the manuscript |
| In your Data Availability statement, you have not specified where the minimal data set underlying the results described in your manuscript can be found. PLOS defines a study's minimal data set as the underlying data used to reach the conclusions drawn in the manuscript and any additional data required to replicate the reported study findings in their entirety. All PLOS journals require that the minimal data set be made fully available.Upon re-submitting your revised manuscript, please upload your study’s minimal underlying data set as either Supporting Information files or to a stable, public repository and include the relevant URLs, DOIs, or accession numbers within your revised cover letter. | We have deposited our data to Zenodo <https://zenodo.org/record/5885191#.YiExfBPMLt0>. The deposited data includes the metadata for all 31 analyzed videos and the code book that describes the variables used. In our manuscript, the data is reference 65. |  |
| Thank you for stating the following in your manuscript:  “Funding was provided by the Social Sciences and Humanities Research Council of Canada (SSHRC) Insight Grant #435-2021-0108 "Metrics Literacies: Improving the understanding and appropriate use of scholarly metrics in academia””  Please remove any funding-related text from the manuscript and let us know how you would like to update your Funding Statement. Currently, your Funding Statement reads as follows:  Please include your amended statements within your cover letter; we will change the online submission form on your behalf. | We have removed the funding statement from the manuscript.  The funding statement that was submitted via Editorial Manager is correct:  “SH and AJ were supported by funds from the Social Sciences and Humanities Research Council of Canada (SSHRC) Insight Grant #435-2021-0108 "Metrics Literacies: Improving the understanding and appropriate use of scholarly metrics in academia” (https://www.sshrc-crsh.gc.ca/)  The funders had no role in study design, data collection and analysis, decision to publish, or preparation of the manuscript.” |  |
| Please review your reference list to ensure that it is complete and correct. If you have cited papers that have been retracted, please include the rationale for doing so in the manuscript text, or remove these references and replace them with relevant current references. | We have reviewed the reference list to ensure that it is complete and correct. We do not cite any retracted papers. |  |
| Reviewer One: |  |  |
| I highly appreciate this new approach to study information dispersion/education, in particular in the field of bibliometrics. | Thank you for these kind words. |  |
| p. 3 the authors’ definition of the h-index does not seem right. They say that a researcher with an h-index of 15 has published at least 15 papers which have received at least 15 citations each. An author having published at least 17 papers which have received at least 17 citations also meets this requirement. I think the authors should add that h is the largest number meeting their requirement. | We have added the clarification that it is the largest number of h papers with at least h citations. | Page 3 |
| p.4 among the problems with the h-index they should mention (already here) the dependence on the database, especially the difference between a Google h-index and a WoS h-index. | We have added a statement that describes the variation between databases and included an example related to discrepancies between Web of Science and Google Scholar. | Page 4 |
| p.11 Although difficult I think that the authors should mention factual errors in these videos, or make a remark when they include statements which are clearly without any nuance or unethical, such as “My professor says...“. | Thank you for this suggestion, however, we respectfully decline to make this change. We have added statements to the manuscript acknowledging this as a limitation of our manuscript and calling attention to the inaccurate statement pointed out by the reviewer. Our hesitation to code for accuracy relates to our inability to make a determination of whether a video was accurate at its time of posting. | Pages 11, 16 |
| Also the statement that publishing in languages other than English is bad for one’s h-index, is besides the point. Publishing in other languages leads to less citations, and in this way influences the h-index. | We have now added a statement that acknowledges that non-English publications have lower citation rates and thus influence the h-index. | Page 12 |
| p.15 A similar remark is valid when talking about gender bias. The h-index is not gender biased as such; citations are. | We have added a statement that recognizes that the concern related to gender bias is related to gender biased citation practices. | Page 15 |
| Some minor remarks  p.2 abstract. Line (-2). I would say a minority (32%) was of professional quality. | We have made this edit. | Page 2 |
| p. 6 line 3. I think that the word ‘thus’ should be removed. | We have removed this word | Page 6 |
| p. 8 video 2. Wrong date | We have rechecked this video, but based on our review it seems that Video 2 is correctly labeled as being posted in 2013. |  |
| A final remark. The authors introduce the notion of “becoming metrics literate” (fine), but although mentioned, they do not state the difference with the term “metric-wise”. In my understanding, becoming metric-wise means becoming metric literate AND being able to use it to one’s advantage. I also note that the term “metric-wise” was discussed in the article mentioned by the authors, but actually introduced in (2015) “Metric-wiseness. JASIST, 66(11), 2389-2389. | We have now added the suggested reference and noted the similarities between the two concepts. | Page 3; References |
| Reviewer Two: |  |  |
| This interesting article studies how videos on YouTube explain and discuss the h-index. After a period in which the metrics community was mostly oriented inwards (e.g., towards developing more sophisticated indicators), it is a welcome change to see increasing attention to the use of metrics by researchers, practitioners, and administrators. The present paper fits into the latter paradigm. | Thank you for your thoughtful comments. |  |
| Overall, I think this paper is very well done, with sufficient attention to various aspects that make a video more or less suitable to learn about the h-index and its limitations. Similar studies could be done on other indicators and platforms, and I expect that that will happen in the coming years. The paper is clearly structured and well-written. I have only some fairly minor suggestions for the authors. | Thank you for your kind words. |  |
| The paper presents solid arguments in favor of studying videos in particular. However, I'd like to point out that there are more options beyond mere text on the one hand and video on the other. For instance, the infographic developed by CWTS (https://leidenmadtrics.nl/articles/halt-the-h-index) presents some drawbacks of the h-index in a static yet visual way. | Thank you for this comment. We agree that materials beyond videos and text can be valuable in conveying information about the h-index. We have added this as a limitation to our study and encourage future researchers to consider these types of resources. | Page 16 |
| Some additional information on the process that led to the final 31 videos would be appropriate. It is not clear to me if these were the first 31 videos presented by YouTube/Google, or if they constitute a truly random sample out of all 274. Table 1 shows that they represent a mixture of older and more recent videos, as well as more and less popular ones (which suggests some kind of randomization) but some more details should be provided. | To clarify our approach, we have added additional details to our methods section. To arrive at the 31 videos, two reviewers each independently watched all 274 videos and considered if they met the study’s inclusion criteria. | Page 6 |
| Can you also describe your findings after reviewing the last 7 videos? Did those yield any new insights or were they all 'more of the same'? | We have added a statement that after reviewing the final seven videos, we did not identify any new insights. | Page 7 |
| I would change the definition of the h-index on p. 3 to read that it is THE LARGEST NUMBER h such that there are h number of papers with at least h citations. (Strictly speaking, someone with h-index 15 also has 10 papers with 10 or more citations but in fact they have even more). | We have added the clarification that it is the largest number of h papers with at least h citations. | Page 3 |
| It would be helpful if the authors could provide some recommendations for future videos on the h-index or other bibliometric indicators: which formats work well? What are issues to be aware of? | Thank you for this suggestion. Based on our findings, we have attempted to make our suggestions more explicit for future video creators, such as optimizing elements related to cognitive load and the integration of caveats about the h-index. However, we do not feel comfortable making more general suggestions as this study was not designed to make specific judgements on best practices and thus it is not possible for us to make specific recommendations about which formats work well or potential issues. We are currently preparing a randomized controlled trial where we want to empirically test what types of formats work best. | Pages 15-16 |
